# Glycemic index, glycemic load, dietary insulin index, and dietary insulin load in relation to cardiometabolic risk factors among participants with atherosclerosis: a cross-sectional study

**DOI:** 10.1186/s40795-023-00755-4

**Published:** 2023-08-15

**Authors:** Hossein Bavi Behbahani, Mahshad Shokuhi, Cain C. T. Clark, Ahmad Zare Javid, Vahideh Aghamohammadi, Hadi bazyar, Mahsa Samadani, Habib Haybar

**Affiliations:** 1 Student Research Committee, Ahvaz Jundishapur University of Medical Sciences, Ahvaz, Iran; 2 Department of Nutrition, School of Allied Medical Sciences, Ahvaz Jundishapur University of Medical Sciences, Ahvaz, Iran; 3 Nutrition and Metabolic Diseases Research Center, Ahvaz Jundishapur University of Medical Sciences, Ahvaz, Iran; 4 Centre for Intelligent Healthcare, Coventry University, Coventry, CV1 5FB UK; 5 Department of Nutrition, Khalkhal University of Medical Sciences, Khalkhal, Iran; 6 Department of Public Health, Sirjan School of Medical Sciences, Sirjan, Iran; 7 Student Research Committee, Sirjan School of Medical Sciences, Sirjan, Iran; 8 Cardiovascular Research Center, Ahvaz Jundishapur University of Medical Sciences, Ahvaz, Iran

**Keywords:** Cardiometabolic risk factors, Dietary insulin index, Dietary insulin load, Glycemic index, Glycemic load

## Abstract

**Background:**

We examined the cross-sectional associations of dietary Glycemic Index (GI), Glycemic Load (GL), Dietary Insulin Index (DII), and Dietary Insulin Load (DIL) with cardiovascular disease (CVD) factors in subjects with atherosclerosis.

**Methods:**

The present cross-sectional study was conducted on subjects with atherosclerosis. Regular dietary intake was assessed using a 168-item semi-quantitative food frequency questionnaire (FFQ) and GI, GL, DIL, and DII were also calculated. Odds Ratio (OR) and 95% Confidence Intervals (CIs) were estimated for general and central obesity according to the GI, GL, DII, and DIL.

**Results:**

According to the continuous score of GL, there was a significant positive association between GL and central obesity for women in all models. Regarding the association between DIL score and biochemical variables, there was a significant positive association between Na and Aspartate transaminase (AST) with DII. Moreover, there was a significant positive association between LDL-c(*p* = 0.03) and AST (*p* = 0.04)with DIL score in all 3 models.

**Conclusion:**

In this study, GL was associated with greater odds of central obesity in women, but not in men. Neither dietary DII nor DIL was associated with BMI and central obesity. GI, GL, DII, and DIL were significantly associated with some CVD risk biomarkers in subjects with atherosclerosis.

## Background

Atherosclerosis, the main pathological process of most CVD, is a long-lasting process beginning in the early decades [1], whilst various factors are associated with the progression of CVD, such as high glucose levels, insulin resistance, high blood pressure, obesity, and dyslipidemia; which are known to be modifiable via alterations in lifestyle and dietary intake [2–5]. Nutritional quality and dietary patterns that influence CVD risk constitute a major target of CVD prevention strategies [6, 7]; however, the optimal dietary pattern to ameliorate the development of atherosclerosis is not well determined [8]. Based on epidemiological studies, a reduction in intake of total fat, particularly saturated fatty acids, is generally agreed [8–10]. Recommendations to reduce fat intake for CVD prevention usually contribute to raised carbohydrate intake [11]; however, carbohydrate-rich diets, per se, have been shown to increase fasting insulin levels versus lower-carbohydrate diets [12, 13]. Chronic hyperinsulinemia is a precursor to obesity, is associated with adipose dysfunctions, and augments meta-inflammation in adipose tissue [14]. The glycemic index (GI), as a measure of carbohydrate quality [13], and Glycemic load (GL), as a measure that incorporates both the quantity and quality of dietary carbohydrates [15], have been associated with increased risk of CVD, stroke, and type 2 diabetes, particularly among overweight individuals via adverse effects on blood lipids and systemic inflammation [15–18]. Findings from the Nurses’ Health Study suggested that high GL was positively associated with CVD risk, and subsequently with hemorrhagic stroke in overweight women [19]. However, the findings of other studies do not support the association of high GI and high GL diet with CVD risk factors [20–23].

Dietary Insulin Index (DII) is a novel algorithm of ranking foods based on their postprandial insulin response to isoenergetic components of foods in comparison to a reference food (analogous to the glycemic index, using either glucose or white bread) [24, 25]. Dietary Insulin Load (DIL) represents another dietary index that is computed by multiplying the DII value of each food by its energy content and the consumption frequency of each food [26]. Bell et al. found an association between adherence to a diet with a high insulin index with improved postprandial glycaemia without an increased risk of hypoglycemia among diabetic patients [27]. Additionally, Nimptsch et al. showed an inverse association between DIL and high-density lipoprotein cholesterol (HDL-c) and a positive association between DIL and triglyceride level, especially in individuals with obesity [28].

Despite the relatively well-accepted association of DII and DIL with several chronic conditions, as seen in earlier studies, to our knowledge, there is no study that has examined the association between DII, DIL, and biochemical indexes among subjects with atherosclerosis. Given the high prevalence of atherosclerosis and its complications, we examined the cross-sectional associations of dietary GI, GL, DII, and DIL with body mass index (BMI), waist circumference (WC), blood lipids, fasting blood sugar, creatinine, blood urea nitrogen (BUN), alanine aminotransferase (ALT), aspartate aminotransferase (AST), Atherogenic index of plasma (AIP), and electrolytes, in subjects with atherosclerosis.

## Methods and materials

### Participants and study design

The present cross-sectional study was conducted on 315 subjects (54% men) with atherosclerosis. In this study, the association between GL, GI, DII and DIL and cardiometabolic risk factors was assessed. We applied clustered random sampling to select subjects referred to four health centers in the Ahvaz Jundishapur University of Medical Science, Iran. Inclusion criteria were; aged 20 years and older, nonimmigrant, having atherosclerosis diseases, having no cancer(s), diabetes, liver, kidney, thyroid, and gastrointestinal disorders, no adherence to specific or prescribed diets, no smoking, no alcohol consumption, no use of any supplements, and no weight changes in the preceding 6 months. We excluded 34 participants for the following reasons; unwillingness to participate in the study, not responding to more than 35 food items in the Food Frequency Questionnaire (FFQ), incomplete demographic or anthropometric data, and reporting a diet with a total energy intake less than 800 kcal and more than 4200 kcal. Demographic and physical activity data were also collected through a questionnaire. The demographic questionnaire included the subjects’ sociodemographic information such as age, marital status, sex, education, economic status, menopause situation (yes or no), duration of menopause (yrs.), coffee consumption, and use of medical pillow; whilst to assess the level of the physical activity of the participants, the short form of the International Physical Activity Questionnaire (IPAQ) was applied through interviewing, and the results were expressed as metabolic equivalent hours per week (METs hr/wk) [29, 30]. The validity of the Persian translation of the short form IPAQ has been confirmed by Dashti et al. (Cronbach’s alpha = 0.7 and test–retest reliability coefficient = 0.9) [31].

### Sample size

The sample size was determined based on BMI (mean = 26.6 and SD = 5.1) as the primary outcome obtained from Hosseinpour-Niazi et al. [32]. The sample size was computed using the following formula: N = [ (z1-α/2)2 × sd2]/d2 (α = 0.05, confidence level of 95% and d = 2%), equating to250 subjects, finally 315 subjects were recruited.

### Dietary assessment

Trained nutritionists obtained the participants’ usual dietary intake through face-to-face interviews. A reliable and valid 168-item semi-quantitative FFQ, with standard servings, was used to assess typical dietary intakes [33]. The consumption frequency of each food item was asked on a daily, weekly, or monthly basis during the past year, and then the portion size of each food reported in household measures was converted to grams per day. Total energy and nutrient intake were then calculated using an adapted version of Nutritionist IV software (the Hearst Corporation, San Bruno, CA), as modified for Iranian foods.

### DII and DIL calculation

Food insulin index refers to the incremental insulin area under the curve over 120 min in response to the intake of approximately a 1000 kJ (239 kcal) portion of the test food, divided by the area below the curve after consumption of a 1000-kJ (239 kcal) portion of the reference food [24]. The insulin index for 68 food items was acquired from previous studies [24, 25, 34]. The insulin index for tea, coffee and salt was considered 0 because the energy and macronutrient content of these foods are near 0. Moreover, for items that were not available in the mentioned food list, the food insulin index of similar items was considered. For example, both dates and raisins are dried fruits. The energy, carbohydrate, fat, protein and fiber contents of both fruits are similar to each other. Thus, insulin index of raisins was used for dates. The average insulin load during the previous year was calculated from the FFQ data by multiplying the insulin index of each food by its energy content per 1 g and the amount of that food consumed (g/d). By summing the insulin load of each food, DIL was obtained for each person; then DII was computed by dividing DIL by total energy intake as follows [35]:

$$\mathrm{Insulin index of food}=\mathrm{ DIL }\div \{\sum [\mathrm{energy content of food }\times \mathrm{frequency of food consumption }\times \mathrm{ serving of food}/\mathrm{day}]\}$$

### GI and GL calculation

Total dietary GI was computed using the following formula [36]:

$$\mathbf{D}\mathbf{i}\mathbf{e}\mathbf{t}\mathbf{a}\mathbf{r}\mathbf{y}\mathbf{G}\mathbf{I}= [(\mathrm{carbohydrate content of food item}) \times (\mathrm{number of servings}/\mathrm{d}) \times (\mathrm{GI of food item})] /\mathrm{total daily carbohydrate intake}$$

$$\mathbf{D}\mathbf{i}\mathbf{e}\mathbf{t}\mathbf{a}\mathbf{r}\mathbf{y}\mathbf{G}\mathbf{L}= (\mathrm{carbohydrate content of each food item}) \times (\mathrm{number of servings}/\mathrm{d}) \times (\mathrm{GI})$$

The GI value of each food item was derived from the international tables of GI [37], the Iranian GI table [38], and the GI online database maintained by the University of Sydney [39]. For foods in which a GI value was not available, they were estimated based on most physically and chemically similar food items. White bread (GI = 100) was considered as the reference food for all derived GI values. As suggested by Willett and Stampfer, we applied the energy-adjusted amount of total carbohydrate intake calculated through the residual method [40].

### Anthropometric assessment

WC, as central obesity index, was measured in a standing position using an anthropometric tape measure, with an accuracy of 1.0 cm, above the iliac crest, just below the lowest rib margin at the end of the regular expiration; whilst for hip circumference, the tape was placed around the point with the maximum circumference over the buttocks [29, 41]. Men with WC > 102 cm and women with WC > 88 cm are at higher risk of obesity disorders than those below these thresholds [42–44]. Bodyweight and height were measured using a Seca scale with an accuracy of 100 gr and a Seca stadiometer with an accuracy of 0.5 cm, respectively; subsequently, BMI, as general obesity index, was computed as the body weight (kg) divided by the square of height (m) [45, 46].

### Biochemical analyses

For biochemical analyses, 5 cc fasting blood samples were drawn from each participant. Fasting blood glucose (FBS), TG (CV interassay = 1.6%), total cholesterol (CV interassay = 2%), HDL-c (CV interassay = 1.8%), and low-density lipoprotein-cholesterol (LDL-c) (CV interassay = 1.29%) were evaluated by the enzymatic method with kits from Pars-Azmoon (Tehran, Iran). Creatinine (Cr) (CV interassay = 3.63%), Blood Urea Nitrogen (BUN) (CV interassay = 5.79%), liver enzymes including ALP (Alkaline phosphatase) (CV interassay = 1.6%), alanine aminotransferase (ALT) (CV interassay = 3.08%), and aspartate aminotransferase (AST) (CV interassay = 4.4%) levels were evaluated in autoanalyser (Roche Cobas Integra 401 plus) using ready to used reagents procured from Roche company. Serum electrolytes such as Na and K were determined by the enzymatic method using kits from Pars-Azmoon (Tehran, Iran) and Autoanalyzer system. The atherogenic index of plasma (AIP) was calculated as the logarithm of the molar ratio of TG/ HDL-C [47].

### Statistical analysis

All statistical analyses were performed using IBM SPSS Statistics software version 24 (IBM SPSS Statistics, Armonk, USA). *P* < 0.05 was, a priori, considered as statistically significant. The normality of variables was evaluated using the Kolmogorov–Smirnov test. Energy-adjusted dietary GI, GL, DII, and DIL scores were categorized into quartiles. The significant differences in variables across the quartiles of GI, GL, DII, and DIL were determined using One-way analysis of variance (ANOVA) for quantitative variables and the chi-square test for qualitative variables. To determine the association between GI, GL, DII, and DIL score with metabolic parameters in atherosclerosis subjects, linear regression analysis was used (3 models; Model 1, linear regression analysis without adjustment; Model 2, linear regression analysis with adjustment for energy intake; Model 3, linear regression analysis with correction for age, sex, energy intake, physical activity, race, BMI, WC, marital status, and education). The odds ratios (95%CI) for general and visceral obesity according to the GI, GL, DII, and DIL score were evaluated using multivariable logistic regression (3 models; Model 1, unadjusted; Model 2, adjusted for energy intake; Model 3, adjustment for age, sex, energy intake, physical activity, race, BMI, WC, marital status and education).

## Results

### The characteristics of participants across quartiles of GI, GL, DII, and DIL

The characteristics of participants across quartiles of GI, GL, DII, and DIL are illustrated in Table 1 and Table 2. The mean age of study participants was 58 ± 11 years, whilst obesity was evident in 26% of men and 28% of women. Those in GI quartile 4 had significantly higher FBS, Cholesterol, LDL-c, Cr, ALT, AST, and AIP compared to those in quartile 1 (*p* < 0.05). Moreover, those in GL quartile 4 had significantly higher values for weight, BMI, WC, FBS, Cholesterol, LDL-c, BUN, Cr, ALT, and AST compared to those in quartile 1 (*p* < 0.05). However, those in GL and GI quartile 1 had significantly higher HDL-c compared to those in quartile 4 (*p* < 0.05). (Table 1).

**Table 1:** The characteristics at baseline across quartiles of GI and GL score

Characteristics (mean (SD) or N (%)	GI quartiles					*P—value*	GL quartiles					*P—value*
	Q1 (*N* = 79)	Q2 (*N* = 78)	Q3 (*N* = 79)	Q4 (*N* = 79)	Total (*N* = 315)		Q1 (*N* = 78)	Q2 (*N* = 80)	Q3 (*N* = 78)	Q4 (*N* = 79)	Total (*N* = 315)	
Score GI—score GL	52.64 ± 3.66	58.81 ± 1.34	62.31 ± 0.94	65.01 ± 1.05	59.70 ± 5.08	** < 0.001**	210.79 ± 37.10	308.11 ± 18.88	368.86 ± 24.94	477.97 ± 38.92	477.97 ± 101.76	** < 0.001**
Age (years)	59.51 ± 10.63	57.44 ± 12.03	59.40 ± 11.19	58.93 ± 11.45	58.75 ± 11.31	0.69	58.85 ± 12.28	60.48 ± 11.24	57.12 ± 10.43	58.50 ± 11.16	58.75 ± 11.31	0.31
Sex (N) (%)						0.07						0.60
Male	37(47)	44(56)	51(64)	37(46)	169(54)		44(56)	41(51)	38(49)	46(58)	169(54)	
Female	42(53)	34(44)	28(36)	42(54)	146(46)		34(44)	39(49)	40(51)	33(42)	146(46)	
Hight (m)	1.66 ± 0.09	1.69 ± 0.10	1.66 ± 0.10	1.67 ± 0.12	1.67 ± 0.10	0.18	1.68 ± 0.11	1.65 ± 0.10	1.65 ± 0.10	1.69 ± 0.10	1.67 ± 0.10	0.13
Weight (kg)	73.65 ± 10.67	77.30 ± 12.90	73.75 ± 13.3	75.55 ± 16.26	75.06 ± 13.46	0.27	72.83 ± 12.05	72.35 ± 14.49	77.64 ± 14.06	77.46 ± 12.38	75.06 ± 13.46	**0.01**
BMI (kg/m2)	26.89 ± 4.44	27.08 ± 4.88	27.00 ± 6.14	27.10 ± 5.86	27.02 ± 5.35	0.99	26.04 ± 51.00	26.33 ± 5.25	28.50 ± 6.09	27.21 ± 4.64	27.02 ± 5.35	**0.01**
BMI categories (N) (%)						0.41						0.12
< 30	61(77)	56(72)	59(75)	52(66)	228(72)		62(79)	58(73)	49(63)	59(75)	228(72)	
≥ 30	18(23)	22(28)	20(25)	27(34)	87(28)		16(21)	22(28)	29(37)	20(25)	87(28)	
WC (cm)	88.72 ± 13.47	93.41 ± 14.84	91.32 ± 16.56	93.10 ± 16.61	91.63 ± 15.46	0.20	88.29 ± 15.38	89.87 ± 15.31	94.46 ± 16.10	93.92 ± 14.43	91.63 ± 15.46	**0.02**
WC categories for female (N) (%)						0.78						0.96
< 102	33(73)	30(73)	25(64)	31(70)	119(70)		25(68)	31(72)	36(69)	27(69)	119(70)	
≥ 102	12(27)	11(27)	14(36)	13(30)	50(30)		12()32	12(28)	14(31)	12(31)	50(30)	
WC categories for male (N) (%)						0.28						**0.05**
< 88	19(45)	11(32)	13(46)	12(29)	55(38)		19(56)	15(38)	13(33)	8(24)	55(38)	
≥ 88	23(55)	23(68)	15(54)	30(71)	91(91)		15(44)	24(62)	27(67)	25(76)	91(62)	
Marital status (N) (%)						0.60						0.36
Single	30(38)	33(42)	36(46)	38(48)	137(43)		29(37)	32(40)	38(49)	38(48)	137(43)	
Married	49(62)	45(58)	43(54)	41(52)	178(57)		49(63)	48(60)	40(51)	41(52)	178(57)	
Educat ion (N) (%)						0.58						0.48
Illiterate	9(11)	1(1)	9(11)	9(11)	28(10)		8(10)	7(9)	7(9)	6(8)	28(9)	
Elementary	25(32)	23(29)	24(31)	24(30)	96(30)		25(32)	20(25)	28(36)	23(29)	96(30)	
Diploma	20(25)	21(27)	15(19)	21(27)	77(24)		17(22)	23(29)	22(28)	15(19)	77(24)	
Bachelor	10(13)	20(26)	16(20)	12(15)	58(18)		18(23)	13(16)	11(14)	16(20)	58(18)	
Master of science	13(16)	11(14)	13(16)	10(13)	47(15)		9(12)	16(20)	8(10)	14(18)	47(15)	
Ph. D	2(3)	2(3)	2(3)	3(4)	9(3)		1(1)	1(1)	2(3)	5(6)	9(3)	
Race						0.92						0.6
Fars	16(20)	18(23)	22(28)	19(24)	75(24)		19(25)	22(28)	18(23)	16(21)	75(24)	
Lore	36(46)	35(45)	35(44)	38(48)	144(46)		30(38)	39(49)	36(46)	39(49)	144(46)	
Arab	27(34)	25(28)	22(28)	22(28)	96(30)		29(37)	19(23)	24(31)	24(30)	96(30)	
P.A (met-min/week)	1262.73 ± 110.19	1248.26 ± 108.82	1252.67 ± 114.53	1225.89 ± 89.63	1247.39 ± 106.57	0.16	1252.42 ± 98.59	1242.30 ± 113.40	1237.69 ± 103.22	1257.15 ± 111.08	1247.39 ± 106.57	0.64
FBS (mg/dL)	99.94 ± 15.98	102.57 ± 17.21	106.87 ± 15.73	104.41 ± 14.15	103.45 ± 15.93	0.04	99.47 ± 17.36	100.92 ± 16.37	102.80 ± 15.71	110.59 ± 11.58	103.45 ± 15.93	** < 0.001**
TG (mg/dL)	135.84 ± 51.11	143.05 ± 66.72	144.62 ± 63.08	155.77 ± 78.03	144.82 ± 65.52	0.29	138.07 ± 71.58	141.33 ± 60.37	137.91 ± 58.40	161.86 ± 69.03	144.82 ± 65.52	0.06
Cholesterol (mg/dL)	188.30 ± 54.71	217.52 ± 76.96	226.92 ± 61.93	221.54 ± 59.77	213.56 ± 65.28	**0.001**	190.12 ± 52.75	201.26 ± 65.16	211.43 ± 70.21	251.25 ± 55.64	213.56 ± 65.28	** < 0.001**
LDL-c (mg/dL)	108.25 ± 39.37	127.60 ± 55.18	131.69 ± 58.52	120.88 ± 58.82	122.09 ± 54.05	**0.03**	99.73 ± 43.42	107.87 ± 52.88	129.20 ± 57.17	151.53 ± 46.94	122.09 ± 54.05	** < 0.001**
HDL-c (mg/dL)	48.32 ± 5.91	49.78 ± 7.20	39.87 ± 7.37	40.27 ± 7.62	44.54 ± 8.35	** < 0.001**	42.33 ± 7.50	45.57 ± 9.30	45.41 ± 8.17	44.84 ± 8.07	44.54 ± 8.35	**0.05**
BUN (mg/dL)	17.20 ± 7.92	16.85 ± 4.70	18.03 ± 4.42	17.07 ± 4.28	17.29 ± 4.59	0.39	15.98 ± 4.63	17.43 ± 4.28	17.92 ± 4.60	17.82 ± 4.66	17.29 ± 4.59	**0.03**
Creatinine (mg/dL)	1.02 ± 0.24	0.97 ± 0.20	1.08 ± 0.21	1.06 ± 0.21	1.03 ± 0.22	**0.005**	0.97 ± 0.19	0.99 ± 0.20	1.07 ± 0.23	1.10 ± 0.22	1.03 ± 0.22	**0.00**
Na (mEq/L)	137.41 ± 3.18	137.41 ± 3.50	136.67 ± 3.60	137.77 ± 3.23	137.31 ± 3.39	0.22	137.37 ± 2.87	137.38 ± 3.32	137.30 ± 3.32	137.20 ± 4.00	137.31 ± 3.39	0.98
K (mEq/L)	4.05 ± 0.46	4.03 ± 0.53	4.11 ± 0.62	4.07 ± 0.47	4.07 ± 0.52	0.81	4.04 ± 0.46	4.07 ± 0.51	4.06 ± 0.62	4.11 ± 0.49	4.07 ± 0.52	0.88
AST (IU/L)	23.93 ± 9.75	27.93 ± 11.74	36.48 ± 13.65	38.18 ± 12.93	31.64 ± 13.40	** < 0.001**	27.64 ± 10.58	28.70 ± 11.16	33.23 ± 13.77	37.02 ± 15.65	31.64 ± 13.43	** < 0.001**
ALT (IU/L)	25.30 ± 11.10	24.51 ± 12.13	32.63 ± 14.14	33.27 ± 12.24	28.94 ± 13.04	** < 0.001**	26.07 ± 9.25	25.11 ± 9.12	31.11 ± 15.58	33.51 ± 15.05	28.94 ± 13.04	** < 0.001**
AIP	0.42 ± 0.17	0.42 ± 0.19	0.52 ± 0.19	0.54 ± 0.22	0.47 ± 0.20	** < 0.001**	0.47 ± 0.21	0.46 ± 0.20	0.45 ± 0.20	0.52 ± 0.20	0.47 ± 0.20	0.08

1. Data are means ± SD for quantitative variables and frequency (percent) for qualitative variables. P-values of statistical significance (p < 0.05) are presented in bold

a. From ANOVA for quantitative variables, b. Chi-square for qualitative variables

Abbreviation: *DII* Dietary insulin index, *DI* dDietary insulin load, *BMI* Body mass index/z, general *WC* Waist circumference, *PA* Physical activity, *FBS* Fasting blood sugar, *TG* Triglyceride, *LDL-c* Low-density lipoprotein cholesterol, *HDL-c* High-density lipoprotein cholesterol, *BUN* Blood urea nitrogen, *Na* Sodium, *K* Potassium, *AST* Aspartate aminotransferase, *ALT* Alanine aminotransferase, *AIP* Atherogenic index of plasma

**Table 2:** The characteristics at baseline across quartiles of DII and DIL score

Characteristics (mean (SD) or N (%)	DII quartiles					*P—value*	DIL quartiles					*P—value*
	Q1 (*N* = 78)	Q2 (*N* = 79)	Q3 (*N* = 79)	Q4 (*N* = 79)	Total (*N* = 315)		Q1 (*N* = 78)	Q2 (*N* = 79)	Q3 (*N* = 79)	Q4 (*N* = 79)	Total (*N* = 315)	
Score DII- score DIL	24.01 ± 6.36	39.54 ± 1.70	42.57 ± 0.54	46.34 ± 3.49	38.16 ± 9.25	** < 0.001**	287.09 ± 57.72	418.90 ± 23.52	513.99 ± 39.51	671.62 ± 54.66	473.49 ± 141.49	** < 0.001**
Age (years)	59.60 ± 11.20	59.79 ± 12.22	58.79 ± 10.94	56.83 ± 10.78	58.75 ± 11.31	0.33	60.28 ± 12.35	58.87 ± 11.97	57.93 ± 10.23	57.94 ± 10.60	58.75 ± 11.31	0.52
Sex (N) (%)						0.79						0.56
Male	43(55)	42(53)	45(57)	39(49)	169(54)		47(60)	41(52)	39(49)	42(53)	169(54)	
Female	35(45)	37(47)	34(43)	40(51)	146(46)		31(40)	38(48)	40(51)	37(47)	146(46)	
Hight (m)	1.67 ± 0.09	1.65 ± 0.10	1.66 ± 0.11	1.69 ± 0.10	1.67 ± 0.10	0.11	1.67 ± 0.11	1.66 ± 0.10	1.66 ± 0.10	1.69 ± 0.10	1.67 ± 0.10	0.24
Weight (kg)	74.16 ± 12.38	75.53 ± 13.31	74.81 ± 12.62	75.73 ± 15.49	75.06 ± 13.46	0.08	71.98 ± 12.43	73.46 ± 13.78	76.65 ± 14 71	78.10 ± 12.11	75.06 ± 13.46	0.10
BMI (kg/m2)	26.48 ± 5.23	27.74 ± 5.53	27.34 ± 5.199	26.50 ± 5.44	27.02 ± 5.35	0.35	25.87 ± 5.30	26.68 ± 4.89	28.04 ± 6.35	27.46 ± 4.55	27.02 ± 5.35	0.06
BMI categories (N) (%)						0.11						**0.02**
< 30	64(82)	52(66)	54(68)	58(73)	228(72)		62(79)	61(77)	47(59)	58(73)	228(72)	
≥ 30	14(18)	27(34)	25(32)	21(27)	87(28)		16(21)	18(23)	32(41)	21(27)	87(28)	
WC (cm)	88.07 ± 14.13	94.06 ± 15.54	93.31 ± 15.75	91.03 ± 15.94	91.63 ± 15.46	0.06	87.82 ± 15.32	91.16 ± 14.31	93.11 ± 16.96	94.39 ± 14.63	91.63 ± 15.46	**0.04**
WC categories for female (N) (%)						0.19						0.26
< 102	30(75)	30(65)	31(62)	28(82)	119(70)		24(69)	31(72)	36(72)	28(68)	119(70)	
≥ 102	10(25)	16(35)	7(38)	17(18)	50(30)		11(31)	12(28)	14(28)	13(32)	50(30)	
WC categories for male (N) (%)						0.35						0.42
< 88	17()	11()	11()	16()	55()		15(48)	13(34)	16(40)	11(30)	55(38)	
≥ 88	18()	26()	23()	24()	91()		16(52)	25(66)	24(60)	26(70)	91(62)	
Marital status (N) (%)						0.9						0.87
Single	32(41)	34(43)	34(43)	37(47)	137(47)		33(42)	32(41)	35(44)	37(47)	137(43)	
Married	46(59)	45(57)	45(57)	42(53)	178(57)		45(58)	47(59)	44(56)	42(53)	178(57)	
Education (N) (%)						0.87						0.77
Illiterate	9(12)	5(6)	9(11)	5(6)	28(9)		10(13)	8(10)	4(5)	6(8)	28(9)	
Elementary	24(31)	20(25)	24(30)	28(35)	96(30)		25(32)	23(29)	27(34)	21(27)	96(30)	
Diploma	14(18)	24(30)	18(23)	21(27)	77(24)		16(21)	22(28)	20(25)	19(24)	77(24)	
Bachelor	15(19)	15(19)	15(19)	13(16)	58(18)		17(22)	14(18)	13(16)	14(18)	58(18)	
Master of science	14(18)	12(15)	12(15)	9(11)	47(15)		10(13)	10(13)	12(15)	15(19)	47(15)	
PhD	2(3)	3(4)	1(1)	3(4)	9(3)		0(0)	2(3)	3(4)	4(5)	9(3)	
Race (N) (%)						0.26						
Fars	16(21)	27(34)	14(18)	19(24)	75(24)		20(26)	17(22)	20(25)	18(23)	75(24)	
Lore	36(46)	30(38)	42(53)	36(46)	144(46)		37(47)	32(40)	36(46)	39(49)	144(46)	
Arab	26(33)	22(28)	23(29)	25(32)	96(30)		21(27)	30(38)	23(29)	220(28)	96(30)	0.79
P.A (met-min/week)	1258.00 ± 111.51	1236.98 ± 92.19	1260.68 ± 110.10	1234.02 ± 110.64	1247.39 ± 106.57	0.26	1262.53 ± 98.28	12.42.41 ± 111.72	1224.00 ± 97.90	1260.79 ± 1114.57	1247.39 ± 106.57	0.07
FBS (mg/dL)	102.76 ± 14.93	105.06 ± 17.31	103.35 ± 14.86	102.63 ± 16.65	103.45 ± 15.93	0.76	100.89 ± 16.19	101.73 ± 16.66	100.40 ± 16.65	110.75 ± 11.62	103.45 ± 15.93	** < 0.001**
TG (mg/dL)	135.80 ± 47.85	161.22 ± 77.34	146.98 ± 65.26	135.17 ± 65.78	144.82 ± 65.52	0.04	140.01 ± 60.82	143.87 ± 64.27	137.17 ± 67.38	158.18 ± 68.51	144.82 ± 65.52	0.18
Cholesterol (mg/dL)	209.32 ± 60.33	219.67 ± 63.82	218.26 ± 66.09	206.93 ± 70.71	213.56 ± 65.28	0.52	200.52 ± 52.66	201.74 ± 68.54	202.73 ± 68.84	249.07 ± 57.21	213.56 ± 65.28	** < 0.001**
LDL-c (mg/dL)	122.25 ± 50.06	125.28 ± 53.85	119.43 ± 53.05	121.38 ± 59.62	122.09 ± 54.05	0.92	100.97 ± 43.33	115.21 ± 54.88	120.58 ± 57.42	151.31 ± 47.29	122.09 ± 54.05	** < 0.001**
HDL-c (mg/dL)	46.73 ± 7.06	43.10 ± 8.11	42.40 ± 9.24	45.98 ± 8.18	44.54 ± 8.35	**0.001**	43.24 ± 8.76	44.89 ± 7.91	43.93 ± 8.07	46.10 ± 8.53	44.54 ± 8.35	0.15
BUN (mg/dL)	17.52 ± 4.58	17.21 ± 4.81	17.73 ± 4.44	16.70 ± 4.54	17.29 ± 4.59	0.52	17.08 ± 4.76	17.30 ± 4.38	17.03 ± 4.43	17.74 ± 4.82	17.29 ± 4.59	0.76
Creatinine (mg/dL)	1.03 ± 0.24	0.98 ± 0.20	1.08 ± 0.22	1.04 ± 0.20	1.03 ± 0.22	0.06	0.98 ± 0.22	1.03 ± 0.22	1.01 ± 0.21	1.11 ± 0.20	1.03 ± 0.22	**0.002**
Na (mEq/L)	136.71 ± 3.23	137.46 ± 3.09	137.22 ± 4.16	137.84 ± 2.91	137.31 ± 3.39	0.20	137.19 ± 2.98	136.79 ± 3.19	137.87 ± 3.27	137.40 ± 3.99	137.31 ± 3.39	0.24
K (mEq/L)	4.00 ± 0.50	4.04 ± 0.48	4.15 ± 0.55	4.09 ± 0.56	4.07 ± 0.52	0.29	4.06 ± 0.44	4.07 ± 0.54	4.01 ± 0.55	4.14 ± 0.56	4.07 ± 0.52	0.47
AST (IU/L)	29.34 ± 11.94	32.45 ± 14.58	33.68 ± 13.34	31.07 ± 13.56	31.64 ± 13.43	0.20	28.35 ± 10.57	29.54 ± 12.17	34.67 ± 14.00	33.97 ± 15.53	31.64 ± 13.43	**0.004**
ALT (IU/L)	26.58 ± 12.33	29.37 ± 11.79	30.91 ± 12.90	28.87 ± 14.80	28.94 ± 13.04	0.21	26.98 ± 10.25	26.51 ± 10.33	32.96 ± 16.37	29.29 ± 13.40	28.94 ± 13.04	**0.007**
AIP	0.44 ± 0.15	0.53 ± 0.22	0.50 ± 0.21	0.42 ± 0.19	0.47 ± 0.20	**0.003**	0.48 ± 0.19	0.47 ± 0.19	0.45 ± 0.22	0.50 ± 0.21	0.47 ± 0.20	0.48

1. Data are means ± SD for quantitative variables and frequency (percent) for qualitative variables. P-values of statistical significance (p < 0.05) are presented in bold

a. From ANOVA for quantitative variables, b. Chi-square for qualitative variables

Abbreviation: *DII* Dietary insulin index, *DI* Dietary insulin load, *BMI* Body mass index/z, general *WC*: Waist circumference, *PA* Physical activity, *FBS* Fasting blood sugar, *TG* Triglyceride, *LDL-c* Low-density lipoprotein cholesterol, *HDL-c* High-density lipoprotein cholesterol, *BUN* Blood urea nitrogen, *Na* Sodium, *K* Potassium, *AST* Aspartate aminotransferase, *ALT* Alanine aminotransferase, *AIP* Atherogenic index of plasma

As shown in Table 2, the differences in WC, TG, HDL, and AIP were significant between DII quartiles (*p* < 0.05). Moreover, those in DIL quartile 4 had significantly higher WC, FBS, Cholesterol, LDL-c, Cr, ALT, and AST, compared to those in quartile 1 (*p* < 0.05). The number of participants with a BMI ≥ 30 kg/m^2^ was significantly higher in DIL quartile 4 compared to those in quartile 1(*p* < 0.05).

### Dietary intake of participants across quartiles of GI, GL, DII, and DIL

The mean ± SD of energy, macronutrient, and food groups at baseline across quartiles of GI, GL, DII, and DIL scores are shown in Table 3 and Table 4.

**Table 3:** The mean ± SD of energy, macronutrient and food groups at baseline across quartiles of GI and GL score

Characteristics (mean (SD) or %)	GI quartiles					P – value^*^	GL quartiles					P – value^*^
	Q1 (*N* = 79)	Q2 (*N* = 78)	Q3 (*N* = 79)	Q4 (*N* = 79)	Total (*N* = 315)		Q1 (*N* = 78)	Q2 (*N* = 79)	Q3 (*N* = 79)	Q4 (*N* = 79)	Total (*N* = 315)	
Energy and macronutrients												
Energy (kcal/d)	3432.13 ± 724.49	3028.78 ± 689.25	2903.09 ± 704.26	2723.79 ± 687.04	3021.92 ± 745	** < 0.001**^a^	2529.83 ± 880.17	2921.45 ± 718.43	2956.44 ± 390.18	3673.75 ± 341.29	3021.92 ± 745.39	** < 0.001**^**a**^
Carbohydrates intake (g)	570.58 ± 141.01	478.58 ± 136.92	440.56 ± 126.66	412.27 ± 114.97	475.37 ± 142.73	0.07	325.00 ± 97.97	444.99 ± 93.69	510.50 ± 101.75	619.90 ± 86.48	475.37 ± 142.73	** < 0.001**
Protein intake (g)	122.61 ± 25.96	104.67 ± 23.76	98.59 ± 25.24	92.27 ± 27.43	104.54 ± 27.43	0.06	75.77 ± 19.39	99.08 ± 19.53	113.02 ± 22.18	130.09 ± 13.95	104.54 ± 27.43	**0.01**
Total fat intake (g)	83.40 ± 25.25	77.49 ± 20.77	85.46 ± 26.94	77.89 ± 24.18	81.07 ± 24.53	0.07	65.74 ± 25.14	77.10 ± 20.20	87.73 ± 25.26	93.65 ± 17.31	81.07 ± 24.53	0.40
Food groups												
Refine grains(g)	634.94 ± 328.31	551.94 ± 205.58	576.77 ± 199.63	594.56 ± 192.33	589.67 ± 239.05	** < 0.001**	312.93 ± 120.39	522.90 ± 114.58	650.48 ± 121.27	870.48 ± 152.32	589.67 ± 239.05	** < 0.001**
Whole grains(g)	186.19 ± 162.93	167.00 ± 86.32	90.44 ± 73.06	29.02 ± 30.26	118.01 ± 118.00	** < 0.001**	91.80 ± 133.55	135.21 ± 135.58	119.20 ± 102.83	125.27 ± 91.34	118.01 ± 118.00	** < 0.001**
Red meat(g)	17.34 ± 8.66	16.28 ± 5.80	17.80 ± 8.69	17.35 ± 6.41	17.20 ± 7.49	**0.009**	13.77 ± 7.41	14.94 ± 5.20	19.06 ± 7.77	21.03 ± 7.03	17.20 ± 7.49	**0.01**
Processed meat(g)	24.43 ± 26.27	17.81 ± 21.56	15.10 ± 17.87	8.04 ± 11.65	16.34 ± 20.79	** < 0.001**	18.03 ± 22.50	16.83 ± 24.28	15.36 ± 18.64	15.14 ± 17.16	16.34 ± 20.79	**0.002**
White meat(g)	48.60 ± 21.13	44.38 ± 14.36	45.23 ± 16.05	45.25 ± 19.50	45.87 ± 18.01	0.64	37.24 ± 17.29	46.56 ± 15.98	47.72 ± 18.65	51.85 ± 17.15	45.87 ± 18.01	**0.01**
Beans(g)	40.52 ± 30.42	32.40 ± 9.66	34.38 ± 10.64	30.19 ± 16.00	34.38 ± 18.96	0.20	35.23 ± 29.53	30.05 ± 8.66	35.53 ± 16.31	36.78 ± 14.85	34.38 ± 18.96	** < 0.001**
Dairy(g)	420.23 ± 155.14	440.19 ± 182.69	455.74 ± 182.15	448.30 ± 171.53	441.12 ± 172.89	** < 0.001**	293.97 ± 126.46	415.75 ± 141.87	502.01 ± 176.93	551.97 ± 121.63	441.12 ± 172.89	** < 0.001**
Vegetable(g)	363.17 ± 167.07	370.44 ± 143.17	378.74 ± 135.55	346.21 ± 131.52	364.62 ± 144.79	0.09	307.35 ± 137.07	333.84 ± 127.37	389.17 ± 142.32	428.10 ± 143.14	364.62 ± 144.79	0.17
Fruit(g)	341 ± 90.70	394.97 ± 125.97	363.84 ± 112.01	307.68 ± 112.61	351.82 ± 114.89	** < 0.001**	285.44 ± 129.42	340.94 ± 89.49	372.04 ± 98.72	408.42 ± 103.39	351.82 ± 114.89	0.62
Sweets(g)	65.15 ± 46.89	34.63 ± 21.97	29.22 ± 18.59	53.66 ± 43.54	45.70 ± 37.83	** < 0.001**	43.70 ± 45.18	37.62 ± 27.72	51.97 ± 38.84	49.66 ± 36.77	45.70 ± 37.83	**0.04**
Nut(g)	14.92 ± 18.06	11.23 ± 21.45	14.64 ± 23.31	7.45 ± 10.88	12.06 ± 19.17	**0.01**	14.37 ± 19.91	11.20 ± 23.00	14.74 ± 21.25	8.00 ± 8.76	12.06 ± 19.17	0.11

The data are presented as “mean ± SD”. *P*-values of statistical significance (*p* < 0.05) are presented in bold

a. The significant difference based on One-way ANOVA without adjustment of energy (*p* < 0.05)

^*^. The significant difference based on Analysis of covariance (ANCOVA) with adjustment of energy (*p* < 0.05)

Abbreviation: *GI* Glycaemic index, *GL* glycemic load

**Table 4:** The mean ± SD of energy, macronutrient and food groups at baseline across quartiles of DII and DIL score

Characteristics (mean (SD) or %)	DII quartiles					P – value^*^	DIL quartiles					P – value^*^
	Q1 (*N* = 79)	Q2 (*N* = 78)	Q3 (*N* = 79)	Q4 (*N* = 79)	Total (*N* = 315)		Q1 (*N* = 78)	Q2 (*N* = 79)	Q3 (*N* = 79)	Q4 (*N* = 79)	Total (*N* = 315)	
Energy (kcal/d)	3432.13 ± 724.49	3028.78 ± 689.25	2903.09 ± 704.26	2723.79 ± 687.04	30.21.92 ± 745.39	** < 0.001**^**a**^	2228.95 ± 577.63	2883.88 ± 491.41	3218.25 ± 544.81	3750.81 ± 380.54	3021.92 ± 745.39	** < 0.001**^**a**^
Carbohydrates intake (g)	535.01 ± 165.35	448.77 ± 139.05	457.72 ± 138.56	460.72 ± 108.08	475.37 ± 142.73	** < 0.001**	85.66 ± 33.62	102.96 ± 27.12	102.99 ± 14.00	126.29 ± 11.88	104.54 ± 27.43	0.12
Protein intake (g)	117.99 ± 30.49	98.11 ± 25.49	100.90 ± 28.19	101.31 ± 20.49	104.54 ± 27.43	**0.01**	381.69 ± 172.41	464.43 ± 137.39	456.97 ± 63.05	597.19 ± 74.48	475.37 ± 142.73	0.10
Total fat intake (g)	88.93 ± 30.93	84.96 ± 22.17	75.81 ± 20.97	74.68 ± 19.98	81.07 ± 24.53	**0.008**	65.09 ± 21.39	80.69 ± 26.20	82.46 ± 21.42	95.84 ± 18.62	81.07 ± 24.53	**0.006**
Refine grains (g)	624.11 ± 300.29	549.98 ± 229.62	621.12 ± 207.80	563.91 ± 201.63	589.67 ± 239.05	** < 0.001**	454.86 ± 269.68	555.60 ± 224.41	578.45 ± 123.60	768.07 ± 202.22	589.67 ± 239.05	0.10
Whole grains (g)	148.04 ± 136.60	79.17 ± 97.38	100.72 ± 117 68	144.47 ± 104.21	118.01 ± 118.00	**0.001**	72.73 ± 104.23	142.33 ± 126.90	99.68 ± 86.14	156.71 ± 131.52	118.01 ± 118.00	**0.02**
Red meat(g)	18.12 ± 9.49	17.25 ± 6.98	16.49 ± 6.15	16.94 ± 7.02	17.20 ± 7.49	0.50	13.44 ± 6.41	17.49 ± 8.56	16.95 ± 5.43	20.86 ± 7.40	17.20 ± 7.49	**0.01**
Processed meat(g)	18.27 ± 18.66	23.42 ± 26.66	9.66 ± 14.41	14.04 ± 19.31	16.34 ± 20.79	< 0.001	20.21 ± 22.15	10.86 ± 17.34	15.80 ± 22.76	18.54 ± 19.67	16.34 ± 20.79	**0.01**
White meat(g)	47.04 ± 20.41	44.45 ± 19.24	46.33 ± 15.71	45.69 ± 16.56	45.87 ± 18.01	0.39	38.56 ± 18.29	46.83 ± 18.43	45.86 ± 16.99	52.13 ± 15.92	45.87 ± 18.01	0.16
Beans(g)	37.21 ± 25.02	35.05 ± 16.74	32.15 ± 15.34	33.15 ± 17.27	34.38 ± 18.96	0.79	27.35 ± 14.57	37.36 ± 21.91	32.48 ± 14.21	40.23 ± 21.42	34.38 ± 18.96	0.06
Dairy(g)	453.02 ± 184.28	413.45 ± 150.80	472.79 ± 174.22	425.36 ± 177.56	441.12 ± 172.89	**0.001**	333.93 ± 162.51	426.43 ± 164.77	468.96 ± 161.26	533.78 ± 141.12	441.12 ± 172.89	**0.001**
Vegetable(g)	366.48 ± 175.71	359.18 ± 138.26	395.54 ± 140.76	337.31 ± 115.03	364.62 ± 144.79	**0.007**	297.37 ± 138.42	360.65 ± 140.82	380.06 ± 134.83	419.55 ± 140.83	364.62 ± 144.79	**0.03**
Fruit(g)	338.27 ± 111.80	381.27 ± 131.91	343.90 ± 111.85	343.67 ± 98.46	351.82 ± 114.89	** < 0.001**	292.80 ± 127.52	330.43 ± 98.16	361.36 ± 87.94	421.96 ± 103.40	351.82 ± 114.89	**0.002**
Sweets(g)	52.05 ± 43.51	37.04 ± 28.21	33.57 ± 21.23	60.21 ± 46.55	45.70 ± 37.83	** < 0.001**	37.57 ± 30.16	41.77 ± 38.25	48.04 ± 36.51	55.31 ± 43.43	45.70 ± 37.83	0.70
Nut(g)	19.64 ± 33.49	12.19 ± 13.10	7.33 ± 5.39	9.18 ± 9.11	12.06 ± 19.17	**0.003**	9.12 ± 11.15	15.11 ± 26.88	13.46 ± 21.21	10.52 ± 12.72	12.06 ± 19.17	**0.04**

The data are presented as "mean ± SD". P-values of statistical significance (*p* < 0.05) are presented in bold

a. The significant difference based on One-way ANOVA without adjustment of energy (*p* < 0.05)

^*^. The significant difference based on Analysis of covariance (ANCOVA) with adjustment of energy (*p* < 0.05)

Abbreviation: *DII* Dietary insulin index, *DIL* Dietary insulin load

Across increasing GI quartiles, participants had lower energy intake, refined grain, whole grain, processed meat, fruit, and nut intake (*p* < 0.05). Whist across increasing GL quartiles, participants had greater intake of total protein, total carbohydrate, refined grain, whole grain, red meat, white meat, beans, dairy and sweets (*p* < 0.05). (Table 3).

As shown in Table 4, those in DII quartile 1 had significantly more energy intake, intake of total carbohydrate, total protein, total fat, refined grain, processed meat, white meat, dairy, and nuts, compared to those in quartile 4 (*p* < 0.05). Across increasing DIL quartiles, participants had higher energy intake, intake of total fat, whole grain, red meat, dairy, vegetable, fruit, and nuts (*p* < 0.05). (Table 4).

### Relationship between GI, GL, DII, and DIL scores and metabolic indices

The associations between GI, GL, DII, and DIL score (independent variables) with metabolic parameters (dependent variables) in atherosclerosis subjects are presented in Table 5. According to quartiles of the GI, there was a significant positive association between GI score and FBS, cholesterol, creatinine, AST, ALT, and AIP in all 3 models, model 1(unadjusted), model 2, and model 3(*p* < 0.05). Regarding the association between GI score and HDL-c, in the all 3 models, there was a significant negative association between these two variables (model 1(unadjusted): β-Coefficients = -0.69 and *p* < 0.001, model 2: β-Coefficients = -0.62 and *p* < 0.001, and model 3: β-Coefficients = -0.64 and *p* < 0.001). Regarding the association between GI score and LDL-c, in the unadjusted model, there was no significant association between these two variables (β-Coefficients = 1.01 and *p* = 0.09). However, in the models 2 and 3, there was a significant positive association between GI and LDL-c (model 1: β-Coefficients = 2.54 and *p* < 0.001, model 2: β-Coefficients = 2.28 and *p* < 0.001).

**Table 5:** The Association between GI, GL, DII, and DIL score (independent variables) with metabolic parameters (dependent variables) in atherosclerosis patients

**Variables**	Model 1^a^			Model 2^b^			Model 3^c^		
	B (Unstandardized)	SE	*P—value*	B (Unstandardized)	SE	*P—value*	B (Unstandardized)	SE	*P—value*
According to GI score									
FBS (mg/dL)	0.42	0.17	0.01	0.73	0.18	< 0.001	0.62	0.18	**0.001**
TG (mg/dL)	1.30	0.72	0.07	1.79	0.76	0.02	1.41	0.79	0.07
Cholesterol (mg/dL)	2.65	0.71	< 0.001	4.26	0.71	< 0.001	3.87	0.72	** < 0.001**
LDL-c (mg/dL)	1.01	0.59	0.09	2.54	0.58	< 0.001	2.28	0.60	** < 0.001**
HDL-c (mg/dL)	-0.69	0.08	< 0.001	-0.62	0.08	< 0.001	-0.64	0.09	** < 0.001**
BUN (mg/dL)	0.04	0.05	0.37	0.08	0.05	0.12	0.10	0.05	0.06
Creatinine(mg/dL)	0.005	0.002	0.05	0.008	0.003	0.002	0.008	0.003	**0.003**
Na (mEq/L)	0.002	0.03	0.96	-0.01	0.04	0.80	-0.009	0.042	0.83
K (mEq/L)	0.005	0.006	0.41	0.004	0.006	0.47	0.006	0.006	0.31
AST (IU/L)	1.05	0.13	< 0.001	1.25	0.14	< 0.001	1.25	0.14	** < 0.001**
ALT (IU/L)	0.78	0.13	< 0.001	0.91	0.014	< 0.001	0.09	0.15	** < 0.001**
AIP	0.01	0.002	< 0.001	0.01	0.002	< 0.001	0.10	0.002	** < 0.001**
According to DII score									
FBS (mg/dL)	-0.01	0.09	0.89	0.11	0.10	0.24	0.04	0.10	0.66
TG (mg/dL)	0.20	0.40	0.61	0.38	0.42	0.36	0.19	0.43	0.65
Cholesterol (mg/dL)	0.08	0.39	0.83	0.73	0.40	0.07	0.41	0.40	0.31
LDL-c (mg/dL)	0.01	0.33	0.97	0.72	0.32	0.02	0.52	0.32	0.11
HDL-c (mg/dL)	-0.10	0.05	0.03	-0.03	0.05	0.52	-0.03	0.05	0.055
BUN (mg/dL)	-0.02	0.02	0.48	-0.005	0.03	0.86	0.006	0.03	0.84
Creatinine(mg/dL)	0.001	0.001	0.44	0.002	0.001	0.08	0.002	0.001	0.10
Na (mEq/L)	0.04	0.02	0.03	0.04	0.02	0.05	0.04	0.02	**0.05**
K (mEq/L)	0.004	0.003	0.21	0.004	0.003	0.24	0.005	0.003	0.14
AST (IU/L)	0.14	0.08	0.07	0.19	0.08	0.02	0.16	0.08	0.06
ALT (IU/L)	0.13	0.07	0.08	0.16	0.08	0.04	0.14	0.08	0.08
AIP	0.001	0.001	0.49	0.001	0.001	0.51	0.00	0.001	0.98

**Variables**	Model 1^a^			Model 2^b^			Model 3^c^		
	B (Unstandardized)	SE	*P—value*	B (Unstandardized)	SE	*P—value*	B (Unstandardized)	SE	*P—value*
According to GL score									
FBS (mg/dL)	0.04	0.009	< 0.001	0.04	0.01	0.01	0.03	0.01	**0.01**
TG (mg/dL)	0.09	0.03	< 0.001	0.15	0.05	0.006	0.11	0.05	**0.04**
Cholesterol (mg/dL)	0.22	0.03	< 0.001	0.23	0.05	< 0.001	0.21	0.05	** < 0.001**
LDL-c (mg/dL)	0.19	0.02	< 0.001	0.15	0.04	< 0.001	0.13	0.04	**0.002**
HDL-c (mg/dL)	0.007	0.005	0.13	-0.02	0.007	0.002	-0.02	0.007	**0.001**
BUN (mg/dL)	0.006	0.003	0.01	0.008	0.004	0.04	0.01	0.004	**0.01**
Creatinine(mg/dL)	0.00	0.00	< 0.001	0.001	0.00	0.001	0.001	0.000	**0.001**
Na (mEq/L)	-0.001	0.002	0.51	0.00	0.003	0.95	0.00	0.003	0.97
K (mEq/L)	0.00	0.00	0.35	0.001	0.00	0.05	0.001	0.00	**0.008**
AST (IU/L)	0.03	0.007	< 0.001	0.06	0.01	< 0.001	0.06	0.01	** < 0.001**
ALT (IU/L)	0.02	0.007	< 0.001	0.05	0.01	< 0.001	0.05	0.01	** < 0.001**
AIP	0.00	0.00	0.02	0.001	0.00	< 0.001	0.001	0.00	**0.002**
According to DII score									
FBS (mg/dL)	0.001	0.00	0.002	0.001	0.00	0.19	0.001	0.00	0.53
TG (mg/dL)	0.00	0.00	0.15	0.00	0.00	0.31	0.001	0.00	0.57
Cholesterol (mg/dL)	0.00	0.00	< 0.001	0.00	0.00	0.03	0.00	.00	0.18
LDL-c (mg/dL)	0.00	0.00	< 0.001	0.00	0.00	0.009	0.00	0.00	**0.03**
HDL-c (mg/dL)	0.001	0.00	0.08	-0.001	0.00	0.43	-0.001	0.00	0.45
BUN (mg/dL)	0.001	0.00	0.74	-0.001	0.00	0.52	-0.001	0.00	0.80
Creatinine(mg/dL)	0.001	0.00	0.006	0.001	0.00	0.10	0.001	0.00	0.12
Na (mEq/L)	0.001	0.00	0.21	0.001	0.00	0.05	0.001	0.00	**0.05**
K (mEq/L)	0.001	0.00	0.39	0.001	0.00	0.20	0.001	0.00	0.10
AST (IU/L)	0.001	0.00	0.01	0.001	0.00	0.01	0.001	0.00	**0.04**
ALT (IU/L)	0.001	0.00	0.08	0.001	0.00	0.08	0.001	0.00	0.16
AIP	0.001	0.00	0.56	0.001	0.00	0.42	0.001	0.00	0.67

a. Model 1, linear regression analysis without adjustment; b. Model 2, linear regression analysis with adjustment for energy intake; c. Model 3, linear regression analysis with correction for age, sex, energy intake, physical activity, race, BMI, WC, marital status, and education

P-values of statistical significance (*p* < 0.05) are presented in bold

Abbreviation: *GI* Glycaemic index, *GL* Glycemic load, *DII* Dietary insulin index, *DIL* Dietary insulin load, *FBS* Fasting blood sugar, *LDL-c* Low-density lipoprotein cholesterol, *HDL-c* High-density lipoprotein cholesterol, *BUN* Blood urea nitrogen, *Na* Sodium, *K* Potassium, *AST* Aspartate aminotransferase, *ALT* Alanine aminotransferase, *AIP* Atherogenic index of plasma

According to quartiles of the GL, there was a significant positive association between GL score and FBS, TG, cholesterol, LDL-c, BUN, creatinine, K, AST, ALT, and AIP in all 3 models, model 1(unadjusted), model 2, and model 3(*p* < 0.05). In the unadjusted model, there was no significant association between GL and HDL-c (β-Coefficients = 0.007 and *p* = 0.13). While in the adjusted models, there was a significant negative association between GL and HDL-c (model 2: β-Coefficients = -0.02 and *p* = 0.002, model 3: β-Coefficients = -0.02 and *p* = 0.001). (Table 5).

Regarding the association between DII score and metabolic parameters, in all 3 models, there was a significant positive association between Na and AST with DII score (*p* < 0.05). LDL-c had a significant association with DII score only in adjusted Model 2 (β-Coefficients = 0.72 and *p* = 0.02). The significant negative association between HDL-c and DII score in the crude model (β-Coefficients = -0.1and *p* = 0.03) disappeared in the adjusted Models. (Table 5).

Regarding the association between DIL score and metabolic parameters, there was a significant positive association between LDL-c and AST with DIL score in all 3 models (*p* < 0.05). Na had a weak association with DIL in the unadjusted model and Model 1(*p* = 0.05). A significant positive association was observed between Cr and FBS with DIL in the crude Model (*p* < 0.05) only. Further, the significant association between Cholesterol and DIL in the unadjusted Model (*p* < 0.011) and Model 2(*p *= 0.030 disappeared in Model 3. (Table 5).

Odds ratios (95%CI) for general and visceral obesity (dependent variables) according to the GI, GL, DII, and DIL score (independent variables among participants) are shown in Table 6. In all subjects, no significant association was observed between GI, GL, and general obesity, and in men no significant association was observed between GI, GL, and central obesity in all models (*p* > 0.05).

**Table 6:** Odds ratios (95%CI) for general and central obesity (dependent variables) according to the GI, GL, DII, and DIL score (independent variables) in atherosclerosis patients

Variable	Or (CI)	B	*P- value	Or (CI)	B	*P- value
According to the percentile of GI				According to the percentile of GL		
General obesity						
Model 1^a^	1.17 (0.93–1.46)	0.15	0.16	1.12 (0.90–1.40)	0.12	0.29
Model 2^b^	1.20 (0.94–1.52)	0.18	0.12	1.26 (0.90–1.77)	0.23	0.16
Model 3^c^	1.07 (0.81–1.40)	0.07	0.60	1.02 (070–1.50)	0.02	0.90
Central obesity for male						
Model 1^a^	1.08(0.81–1.45)	0.08	0.58	0.97(0.71–1.33)	-0.02	0.88
Model 2^b^	1.05(0.77–1.44)	0.05	0.71	1.08(0.70–1.67)	0.08	0.71
Model 3^c^	1.07(0.77–1.49)	0.07	0.65	1.11(0.70–1.77)	0.11	0.64
Central obesity for female						
Model 1^a^	1.19(0.98–1.58)	0.17	0.22	1.55(1.12–2.15)	0.44	**0.008**
Model 2^b^	1.35(0.98–1.87)	0.30	0.06	1.69(1.09–2.63)	0.53	**0.018**
Model 3^c^	1.31(0.91–1.90)	0.27	0.13	1.93(1.13–3.28)	0.65	**0.015**
According to the percentile of DII				According to the percentile of DIL		
General obesity						
Model 1^a^	1.12 (0.89–1.40)	0.11	0.30	1.19 (0.95–1.49)	0.18	0.11
Model 2^b^	1.13 (0.90–1.43)	0.13	0.26	1.27 (0.97–1.67)	0.24	0.07
Model 3^c^	1.05 (0.80–1.38)	0.05	0.70	1.16 (0.85–1.58)	0.15	0.33
Central obesity for male						
Model 1^a^	1.11(0.83–1.50)	0.11	0.45	1.00(0.74–1.37)	0.007	0.96
Model 2^b^	1.10(0.80–1.49)	0.09	0.54	1.06(0.75–1.52)	0.06	0.71
Model 3^c^	1.13(0.81–1.55)	0.12	0.45	1.12(0.77–1.62)	0.11	0.54
Central obesity for female						
Model 1^a^	1.09(0.81–1.46)	0.08	0.57	1.22(0.90–1.67)	0.20	0.19
Model 2^b^	1.17(0.85–1.60)	0.15	0.32	1.14(0.80–1.62)	0.13	0.46
Model 3^c^	1.12(0.78–1.60)	0.11	0.52	1.01(0.68–1.51)	0.01	0.93

^*^ *P* < *0.05* statistically significant by Multivariable Logistic Regression. **a**. Model 1: unadjusted. **b**. Model 2: adjusted for energy intake. **c**. Model 3: adjustment for age, sex, energy intake, physical activity, race, BMI, WC, marital status and education

*P-*values of statistical significance (*p* < 0.05) are presented in bold

Central obesity: obese for female (WC ≥ 88 cm) or normal (WC < 88), obese for male (WC ≥ 102 cm) or normal (WC < 102)

General obesity: normal (BMI < 30) or obese (BMI ≥ 30)

Abbreviation: *GI* Glycaemic index, *GL* Glycemic load, *DII* Dietary insulin index, *DIL* Dietary insulin load

According to the continuous score of GL, there was a significant positive association between GL and central obesity for women in all 3 models: model 1 or unadjusted model, model 2, and model 3 (OR = 1.55 (CI: 1.12–2.15), OR = 1.69 (CI: 1.09–2.63), and OR = 1.93 (CI: 1.13–3.28), respectively. A positive association was found in all models between DII and DIL with central obesity, but it was not statistically significant in either gender (*p* ≥ 0.05). (Table 6).

## Discussion

To our knowledge, this is the first study in which body mass index (BMI), waist circumference (WC), blood lipids, fasting blood sugar, creatinine, blood urea nitrogen (BUN), alanine aminotransferase (ALT), aspartate aminotransferase (AST), Atherogenic index of plasma (AIP), and electrolytes were evaluated to provide better insight into the association between dietary GI, GL, DII, and DIL and cardiometabolic risk factors in subjects with atherosclerosis. According to the continuous score of GL, there was a significant positive association between GL and central obesity for women in all models. Regarding the association between DIL score and biochemical variables, there was a significant positive association between Na and AST with DII. Moreover, there was a significant positive association between LDL-c and AST with DIL score in all 3 models.

### Relationship between GI, GL, DII, and DIL scores and obesity

In the present cross-sectional study, we found that a high dietary GL was associated with greater odds of central obesity in women, but not in men. We failed to detect any significant association between DII, and DIL, and central obesity, as well as general obesity, in either gender. Associations between dietary GL, GI, DII, and DIL and central obesity have been addressed in only a relatively small number of studies, and results are equivocal. In the present study, a high dietary GL was associated with greater odds of central obesity in women. Concordant with our results, findings from a study conducted in the UK revealed a significant association between dietary GL and central obesity [48]. In contrast, however, in a cross-sectional study with 5219 subjects, a positive association between GI and abdominal obesity was observed, but not regarding GL [49]. Similar to our observations, in some other research studies that were conducted in Japan, Spain, and the USA, respectively, no significant associations between GI and BMI and central obesity were found [22, 50, 51]. However, findings from the Danish arm of the Monitoring Trends and Determinants in Cardiovascular Disease (MONICA) project showed that adherence to a high-GI diet was associated with higher body weight, body fat, and waist circumference in women, but not in men [52]. It is unknown why women, but not men, appear to be sensitive to the adverse effects of a high dietary GI, particularly for obesity development, but it has been suggested that women may be more susceptible to the adverse effects of a high GI diet than men [49, 52]. Indeed, it is known that fat accumulation might be affected by gonadal steroids that can increase the hypothalamic expression of the orexigenic peptides such as neuropeptide Y and agouti-related peptide [53, 54]. Moreover, high plasma adiponectin levels in females compared to males might be another reason for this sex-mediated discrepancy [26, 55].

The mechanisms pertaining to how dietary GI might affect central and general obesity are largely unclear. Although, it has been posited that high-GI diets may promote excessive food intake and subsequent overeating [56]. Indeed, a low-GI diet is considered the most effective diet for the prevention of obesity by lowering postprandial blood glucose and insulin levels, increase satiety, and decrease energy intake [49, 57].

In the present study, DII and DIL were not associated with BMI and waist circumference. However, it has been demonstrated that a diet with high insulinemic potential, due to enhancing insulin secretion during a long period, can reduce fat oxidation and increase carbohydrate oxidation resulting in the development of fat mass and increasing the risk of obesity [58, 59]. Indeed, these observations were correspondent with earlier results, including a prospective study conducted by Joslowski et al. [58], a cross-sectional study by Mozaffari et al.[35], and a case–control study by Anjom-shoae et al. [60]. Joslowski et al. suggested a prospective negative effect of dietary insulin demand during puberty on the body fat percent in young adulthood. The authors concluded that postprandial increases in insulinemia rather than increases in glycemia can induce an undesirable development of body composition [58] and Anjom-Shoae et al. found no significant association between DIL, DII, and odds of abdominal obesity [60]. In contrast, in a cross-sectional study by Sadeghi et al., DIL and DII had a positive association with odds of metabolic syndrome [26]. The inconsistency present in the literature might be due to different tools used for dietary assessment, different eating habits, different food processing and cooking methods in different cultures, different age ranges used, considering different confounder variables, and different methods for computing DIL and DII values.

### Relationship between GI, GL, DII, and DIL scores and biochemical indices

In linear regression analysis, according to quartiles, we found a significant association between GI score and FBS, cholesterol, HDL-c, LDL-c, creatinine, AST, ALT, and AIP in adjusted models. We observed that GL was significantly associated with FBS, TG, cholesterol, LDL-c, BUN, creatinine, K, AST, ALT, and AIP in the adjusted models.

Regarding the association between DIL score and biochemical variables, there was a significant positive association between Na and AST with DII, whilst DIL was significantly associated with LDL-c and AST.

To our knowledge, this is the first study to have evaluated the association of GI, GL, DII, and DIL, and liver enzymes. Indeed, there is growing evidence that diet and nutrients can influence the pathophysiology of non-alcoholic fatty liver disease (NAFLD); where the fatty liver overproduces the liver enzymes ALT, AST, and gamma-glutamyltransferase (GGT), C-reactive protein (CRP), and coagulation factors. Overfeeding either saturated fat or carbohydrate increases liver fat content [61], and dietary carbohydrates, particularly sugars, are known to contribute to elevated blood insulin and TG levels and cause increased hepatic de novo lipogenesis and decreased hepatic insulin sensitivity, due to the lipogenic potential of fructose during liver metabolism [62].

In the present study, we also found that dietary GI and GL were positively associated with fasting blood glucose after adjustment for confounding factors. In a large cohort of young women, those in the highest quintile of dietary GI (high in rapidly absorbed carbohydrates and low in cereal fiber) had a higher risk of diabetes versus the lowest [63]. Moreover, in the Tehran Lipid and Glucose Study, dietary GL, but not GI, had a positive association with plasma FBS and 2-h blood glucose levels, only among nonobese subjects [32]. A cross-sectional analysis of the Saku Control Obesity Program study reported that high dietary GL was positively associated with greater odds of HbA1c ≥ 7.0% [64]. However, some cross-sectional studies did not show any association between dietary GI and FBS [64–66] and 2-h blood glucose concentrations [64].

In the current study, consistent with a cross-sectional setting and with the use of data from the Nurses’ Health Study and the Health Professionals Follow-Up Study, DII and DIL were not associated with fasting biomarkers of glycemic control [28]. However, in another cross-sectional study, DIL had a positive association with serum levels of FBS in elderly men [35]. It is apparent that prolonged intake of foods with high DII can lead to β-cell dysfunction; indeed, this condition can subsequently lead to insulin resistance and elevated blood FBS concentrations [28].

Regarding lipid profile, the adjusted concentrations of cholesterol, TG, and LDL-c increased across the quartiles of dietary GL while there was no significant correlation between GL and HDL-c concentration. Also, dietary GI was associated with blood HDL-c, LDL-c, and cholesterol levels. These findings corroborate results from several previous studies [12, 18, 32, 50, 67, 68]. In a meta-analysis of prospective studies, high dietary GI was related to an increased risk of CHD [69]. The association of dietary GI and dietary GL with CVD is stronger in overweight than in normal-weight subjects in prospective studies [69–71]. However, the relationship of dietary GI or GL with cardiometabolic risk factors remains unresolved [22, 23].

DIL had a positive association with LDL-c concentrations, howeever we found no association between DIL and DII and other blood lipid variables. In contrast, Nimptsch et al. found an inverse association between DIL and HDL-c and a positive association between DIL and TG. It appears that the association of DIL with TG and HDL-c indicated by Nimptsch et al., particularly in participants with obesity, may be due to the elevated insulin resistance in this group [28]. This suggests that the dietary insulin index would be applicable in heavier subjects, whilst the mean BMI of our participants was approximately 27.02 kg/m^2^ (in the overweight BMI classification). Another putative reason for why we failed to detect any association between DIL, DII, and CVD risk biomarkers such as HDL-c, TG, FBS, and total cholesterol, might be due to the overall low mean DII and DIL in our study (38.16 ± 9.25 and 473.49 ± 141.49, respectively). Further, due to our study's cross-sectional design, inferring a causal link between GI, GL, DIL, DII, and CVD was precluded. Hence, studies with prospective designs are required to confirm these relationships over longer periods. A low sample size and Lack of data pertaining to the medication and the duration of the disease, postprandial levels of glucose, Insulin, HbA1C, and other cardiometabolic risk factors were another limitation of the present study. Moreover, Applying the FFQ for evaluating dietary assessment, is another of the limitations of our study due to it depends on long-term memory and is not accurate enough.

However, despite the aforementioned limitations, there were several strengths that merit acknowledgement. Indeed, the inclusion of both sexes in the study, using linear regression analysis and multivariable logistic regression in 3 different models, and the use of a validated FFQ for regular dietary assessment provided a robust platform to interrogate the incumbent data. Additionally, we considered recent changes in body weight and dietary habits as inclusion criteria, as they have been appeared to be associated with cardiometabolic risk factors. Finally, another strength of this work is its’ novelty, where, to our knowledge, this is the first study in which body mass index (BMI), waist circumference (WC), blood lipids, fasting blood sugar, creatinine, blood urea nitrogen (BUN), alanine aminotransferase (ALT), aspartate aminotransferase (AST), Atherogenic index of plasma (AIP), and electrolytes were evaluated to provide better insight into the association between dietary GI, GL, DII, and DIL and cardiometabolic risk factors in subjects with atherosclerosis.

## Conclusions

In this cross-sectional study, GL was associated with greater odds of central obesity in women, but not in men. Neither dietary DII nor DIL was associated with BMI and central obesity, whilst GI, GL, DII, and DIL had significant associations with some CVD risk biomarkers in subjects with atherosclerosis. Future studies with prospective and interventional designs are needed to clarify the association between these dietary indices and cardiometabolic risk factors among subjects with atherosclerosis.

## Data Availability

The datasets analyzed in the current study are available from the corresponding author on reasonable request.
